# Hot versus cold subduction initiation

**DOI:** 10.1093/nsr/nwae012

**Published:** 2024-01-08

**Authors:** Zhong-Hai Li

**Affiliations:** Key Laboratory of Computational Geodynamics, College of Earth and Planetary Sciences, University of Chinese Academy of Sciences, China

## Abstract

Initiation of a new subduction zone could act in two different ways, forming either a hot or cold incipient subduction channel with contrasting geological records.

In the framework of plate tectonics, subduction initiation (SI) indicates the birth of a plate convergent boundary, which is a key point of plate tectonics and Wilson's cycle; however, its dynamic process is not well constrained and thus remains a controversial issue. Comparing to the long-term mature subduction, its initiation is more like an ‘instantaneous’ process with limited geological records. Furthermore, these records experience erosion and modification by the later subduction. Consequently, the remnant geological records are rare, which is a major barrier for the better understanding of the SI process.

Most of the previous investigations focus on the modes and driving forces of SI by the primary method of geodynamic modeling [1,2]. However, the final solution of the SI mystery depends on the geological records in nature, including the magmatic, metamorphic and structural-sedimentary responses. The most widely studied characteristic magmatic record is the forearc rock sequence (forearc basalt—boninite—arc tholeiites) in the Izu-Bonin-Mariana (IBM) subduction zone [3]. In addition, the SSZ-type (Supra-Subduction-Zone) ophiolites, e.g. in the Troodos (Cyprus) and Semail (Oman), have comparable petrological and geochemical characteristics with the IBM forearc sequence [3]. Thus, it is further proposed that the SSZ-ophiolite could be generated during the SI. Such magmatic responses, i.e. the generation of IBM forearc sequence or SSZ-ophiolite, generally require a high temperature condition in order for the partial melting and melt extraction in the shallow depth, similar to the decompression-induced partial melting in the mid-ocean ridge. The relevant numerical models indicate that the vertically driven slab sinking, accompanying asthenosphere upwelling, plays a critical role in forming such kind of rock records during SI [4].

Another type of geological record for SI is the metamorphic sole, which normally emplaces accompanying with the SSZ-ophiolite. The soles discovered in the present Earth are mostly generated under a low pressure of 0.5–1.5 GPa and high temperature of 700–900°C [5]. Such a critical condition generally requires heating from the asthenosphere, by means of asthenospheric upwelling or greatly thinned overriding lithosphere [6]. The related numerical models indicate that induced SI beneath a rather thin overriding plate contributes to the generation of pressure-temperature conditions of most metamorphic soles [7]. All the aforementioned magmatic and metamorphic records point to a high temperature and low pressure condition for SI. Then, the question is whether the occurrence of all the SI in nature requires such a critical condition with rather high temperature at shallow depths.

In the present-day ocean, there are several early-stage subduction zones with differential geological records. For example, the Puysegur subduction zone to the south of New Zealand first initiated at about 16 Ma in the northern segment and then propagated to the south [8]. This SI is controlled by the transpressional tectonic forces with both compression and strike-slip components, which is a favorable condition for SI. The strong coupling of converging plates under transpression does not allow asthenosphere upwelling and heating; thus, the high temperature and low pressure condition for ophiolite generation and metamorphic sole exhumation cannot be achieved. Consequently, this SI process lacks the typical magmatic and metamorphic records. Instead, the previous studies focus on the responses of structural deformation and sedimentary evolution [9]. Similarly, there are a series of young oceanic subduction zones in the western Pacific, e.g. the Negro subduction zone in the Sulu Sea, the north Sulawesi and Cotabato subduction zones in the Celebes Sea. Their SI is mostly driven by regional compression and experiences the passive underthrusting at the initial stages [10]. In addition, the terrane collision-induced SI may have occurred repeatedly under the long-term plate convergence during the Tethyan evolution [11]. The thermal conditions in these incipient subduction channels should be colder, at least lower than the required temperature for the generation of ophiolite and metamorphic sole.

Based on the above analyses, it indicates that the extremely high temperature condition at shallow depths, for the generation of naturally observed ophiolite and metamorphic sole, only represents the high temperature end-member of SI, but cannot be used as the diagnostics for all the SI. I further propose two contrasting regimes for SI, i.e. the hot versus cold end-members, as shown in Fig. 1.

**Figure 1.**
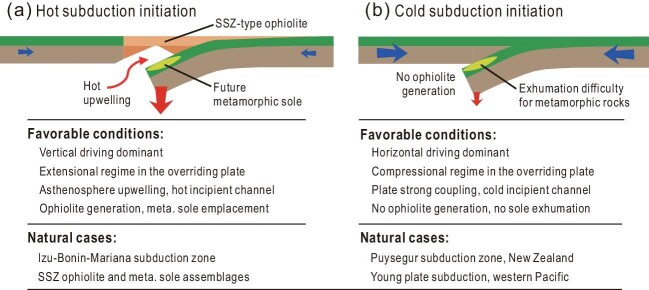
Comparisons between hot and cold subduction initiation.

The hot SI regime is more ‘traditional’ (Fig. 1a), with the geological records of SSZ-ophiolite and metamorphic sole which have been regarded as the typical responses of SI and even as the diagnostics for deciphering paleo-SI cases in the orogens. The high temperature condition at shallow depths generally requires heating from the asthenosphere. The SI driven by dominant vertical forces is more favorable for obtaining such conditions, although the horizontally forcing SI is also applicable when the overriding lithosphere is rather thin [6,7].

The cold SI regime (Fig. 1b) is generally discussed in geodynamic models, but attracts less attention in observational studies, because it lacks relevant magmatic and metamorphic records. In the present Earth, this type of SI generally occurs with young subducting plates which have low negative buoyancy. Thus, the horizontal pushing is the main driving force for SI. In this regime, the relatively strong coupling between converging plates is expected. The overriding plate is under compression during the SI, which could be accompanied with thrust faulting deformation. After the maturation of subduction, the compression state may change to extension with relevant sedimentary responses, e.g. as recorded in the Puysegur subduction zone [9].

The new classification of hot versus cold SI focuses on the geological records, whereas the old ones (spontaneous versus induced SI in [12]; vertical versus horizontal driving in [1]; and so on) focus on the types of driving forces. The new hot SI regime (Fig. 1a) favors vertical driving, but does not exclude the horizontal driving cases. As a typical example, the tectonic reversal of a spreading ridge may lead to the occurrence of SI. Due to the general buoyancy of the ridge, its SI could be driven by the horizontal compression; but I think such type of SI should be categorized into the hot SI regime, because the incipient subduction channel is hot and can generate the metamorphic sole under high temperature conditions [6]. On the other hand, the new cold SI regime favors horizontal driving, but does not exclude the vertical driving cases. In other words, the cold SI regime could also be achieved by vertical driving forces if coupling of converging plates is obtained.

As a conclusion, the SI could occur in contrasting regimes with multiple driving forces. As two end-members, the hot versus cold SI regimes generate different geological records. The SSZ-ophiolite and metamorphic sole are the typical records of hot SI, but are not necessarily generated in the cold SI regime. Thus, we cannot use such a specific rock sequence record to judge the occurrence of SI or not; instead, multiple geological responses should be combined together to get a full view of this puzzling issue.
